# Catholic Knights of America

**Published:** 1881-11

**Authors:** 


DR. E. MILES WILLETT, of Memphis, Tenn., was elected Supreme Medical Director of the Catholic Knights of America, at Louisville, recently.

